# Second Primary Lung Cancer After Breast Cancer: A Population-Based Study of 6,269 Women

**DOI:** 10.3389/fonc.2018.00427

**Published:** 2018-10-09

**Authors:** Rong Wang, Zhiqiang Yin, Lingxiang Liu, Wen Gao, Wei Li, Yongqian Shu, Jiali Xu

**Affiliations:** ^1^Department of Oncology, The First Affiliated Hospital of Nanjing Medical University, Nanjing, China; ^2^Department of Dermatology, The First Affiliated Hospital of Nanjing Medical University, Nanjing, China

**Keywords:** breast cancer, lung cancer, second primary, TNBC, SEER, population-based study

## Abstract

**Purpose:** Breast cancer (BC) and lung cancer are the most two common cancers with highest morbidity and mortality for women. With prolonged survival, there comes the possibility that BC patients will develop second primary cancers. We evaluate the characteristics, incidence and survival of second primary non-small cell lung cancer (BC-NSCLC) and small cell lung cancer (BC-SCLC) after breast cancer.

**Patients and methods:** Second primary lung cancer risks using standardized incidence ratios (SIRs) [95% confidence intervals (95% CIs)] were calculated among breast cancer patients in SEER-18 (2000–2014). Survival outcomes were also analyzed for both BC-NSCLC and BC-SCLC.

**Results:** A total of 6,269 second lung cancer patients after a localized or regional BC were identified. The incidence rate was modestly higher compared to the general population (SIR = 1.03; 95%CI: 1.00–1.06). For ER-, PR- and HER2- groups, SIRs were 1.26, 1.16, 1.13, respectively (all *p* < 0.05). Triple negative breast cancer (TNBC) patients have an even higher incidence rate of lung cancer (SIR = 1.59, 95%CI: 1.29–1.94). Elevated SIRs were also observed among the following groups: within 1 year after BC diagnosed, a young age at BC diagnosed, black people, poorly or undifferentiated histological grade of breast cancer. Median survival (MST) after localized, regional and distant BC-NSCLC was 68.0, 26.0, and 6.0m. Five-year survival rates for BC-NSCLC were 53.9, 29.8 and 5.7% in each stage, which were significantly higher compared to first primary NSCLC (all *p* < 0.001). ER-/PR- or TNBC were unfavorable prognostic factors for BC-NSCLC. The survival rates of BC-SCLC were no significant different compared to first primary SCLC.

**Conclusion:** BC patients, especially for TNBC, are at a high risk of developing second primary lung cancers. BC history may be a favorable prognostic factor for NSCLC (but not SCLC) patients. Clinicians should closely follow up BC patients with high-risk factors.

## Introduction

Breast cancer is the most frequently diagnosed malignancy in women worldwide. During the last three decades, advances in early detection and treatment have resulted in significantly improved survival benefit among BC patients (1–3). The long life expectancy of these patients exposes them to the possibility of developing second primary cancers (4). By 10 years after BC diagnosis, approximately 10% of survivors have developed a subsequent malignancy, in which lung cancer accounts for one of the largest numbers (5). A population-based study from Taiwan demonstrated that second primary cancers indeed have a negative impact on survival in breast cancer patients (6).

Lung cancer is the most common cause of cancer death. Thus, understanding the risk of BC patients developing second primary lung cancer becomes very important. Among women diagnosed with BC before age of 50 years, the incidence rate of lung cancer is significantly elevated (7). Also, postmastectomy radiotherapy for BC patients could sharply increase the risk of second primary lung cancer, especially in ipsilateral lung, among ever-smokers (8). However, no large population study was carried out. The extent to which previous BC confers an increased risk of lung cancer remains unclear. Even few studies focused on the subsequent survival. One study of outcomes in BC survivors with second lung cancer included 35 patients, and another included 26 patients (9, 10). Only one large population-based study compared survival outcomes of BC-NSCLC to those with first primary NSCLC (11). BC history does not appear to adversely affect overall survival (OS). Moreover, no study carried out the survival analysis of second primary SCLC after BC.

Identification the risk factors of developing second lung cancer for BC patients and which factors impact their outcomes are important for identifying those who would benefit from enhanced screening and optimizing treatment. In this study, we conducted a population-based study using data from the Surveillance Epidemiology, and End Results (SEER) Program to evaluate the characteristics, risk and survival of second primary lung cancer after BC.

## Materials and methods

### Ethical statement

This study was approved by the institutional review board of Nanjing Medical University. The data released from SEER database did not require informed patient consent because cancer is a reportable disease in the US.

### Study population

The SEER 18 Program (2000–2014, based on November 2016 data submission) database was used to identify the cohort of women for this study. Largest geographic coverage is available in the SEER 18 database, which capture ~27.8% of the US population. We selected patients ages 20 years and older with a histologically confirmed first primary breast cancer and a subsequent lung cancer between January 2000 and December 2014, excluding those who were diagnosed by autopsy or death certificate only. A minimum latency of 2 months was required, as used in SEER to exclude synchronous primary cancers (12). In order to minimize the possibility of misdiagnosed metastases, we excluded BC cases that were not diagnosed at local or regional stage. This staging is described in detail in historic SEER coding manuals. Male patients were excluded because only 55 cases met the criteria. Information on age at diagnosis, race, and details about BC and lung cancer, as well as latency between diagnoses was obtained from SEER. Because SEER began collecting human epidermal growth factor receptor 2 (HER2) status since 2010, the analyses about HER2 and Breast cancer subtype did not include BC cases diagnosed before 2010. Breast cancer histology was categorized into 2 groups by ICD-O-3 histology codes: invasive ductal carcinoma (IDC, 8500/3, and 8521/3) and others. Lung cancer histology was categorized into 4 groups by ICD-O-3 histology codes: adenocarcinoma (8410, 8250-8253, 8255, 8260, 8323, 8480-8481, 8550, 8560, 8570, 8574), squamous cell (8070-8073, 8083), other NSCLC, and small cell (8041-8045).

### Statistical analysis

The incidence of second primary lung cancer was compared to the expected incidence in the general population by calculating standardized incidence ratio (SIR) and 95% confidence interval (CI). SIRs were calculated overall and stratified by clinical parameters. Frequency distributions of variables were compared using chi-square test. Overall survival (OS) and the association with variables were estimated using Kaplan-Meier methods and log-rank test. Survival times were measured from date of lung cancer diagnosis until date of all-cause death or last follow-up. *T* test was used to compare the survival rates between second and first primary lung cancers. SIRs and survival for first primary NSCLC and SCLC (NSCLC-1 and SCLC-1) were calculated using the SEER^*^Stat software version 8.3.4 and all other analyses were done using SPSS statistical software, version 23 (IBM Corp, Armonk, NY). All *p* values were two-sided, with *p* < 0.05 defined as statistically significant.

## Results

A total of 620,429 female patients were diagnosed with a localized or regional BC in the SEER 18 database between 2000 and 2014. The characteristics of these patients are described in Table 1. One percent or 6269 patients developed a second primary lung cancer and were observed with 3,631,452 person-years of follow-up. The median latency period between breast cancer diagnosis and second lung cancer was 49.0 months.

**Table 1:** SIRs for second primary lung cancer in breast cancer patients diagnosed between 2000 and 2014 with follow-up through 2016, SEER-18.

**Characteristic**	**No. of first primary breast cancers^a^**	**No. of person-years^b^**	**Second primary lung cancers**			
			**Observed**	**Expected**	**SIR**	**95% CI**
Total	620,429	3,631,452	6269	6090.30	1.03^*^	1.00–1.06
**RACE**						
White	503,423	2,996,701	5423	5380.86	1.01	0.98–1.04
Black	63,541	336,635	557	473.50	1.18^*^	1.08–1.28
Other^c^	50,141	281,619	289	211.05	1.37^*^	1.22–1.54
**LATENCY**						
≤ 1 year	620,429	489,378	958	731.65	1.31^*^	1.23–1.39
>1 year, ≤ 5 years	559,541	1,751,111	2656	2805.31	0.95	0.91–0.98
>5 years, ≤ 10 years	329,247	1,099,837	2046	1971.50	1.04	0.99–1.08
>10 years	127,417	291,125	609	581.84	1.05	0.97–1.13
**AGE AT BREAST CANCER DIAGNOSIS**						
20–39	35,216	217,949	44	18.31	2.40^*^	1.75–3.23
40–49	117,093	750,985	387	286.54	1.35^*^	1.22–1.49
50–59	157,050	984,446	1152	1067.37	1.08^*^	1.02–1.14
60–69	146,593	844,699	2156	2074.73	1.04	1.00–1.08
70–79	104,849	581,919	1943	1948.01	1.00	0.95–1.04
80+	59,569	251,086	587	695.34	0.84^*^	0.78–0.92
**YEAR OF DIAGNOSIS**						
2000–2004	202,766	1,900,240	3353	3371.98	0.99	0.96–1.03
2005–2009	206,356	1,274,265	2143	2037.13	1.05^*^	1.01–1.10
2010–2014	211,307	456,946	773	681.19	1.13^*^	1.06–1.22
**STAGE AT BREAST CANCER DIAGNOSIS**						
Localized	414,442	2,471,179	4534	4401.97	1.03^*^	1.00–1.06
Regional	205,987	1,160,272	1735	1688.32	1.30	0.98–1.08
**LATERALITY**^d^						
Right	305,425	1,789,340	3078	2989.69	1.03	0.99–1.07
Left	314,654	1,840,452	3188	3097.50	1.03	0.99–1.07
**HISTOLOGY**						
Infltrating ductal carcinoma	453,626	2,613,812	4430	4233.77	1.05^*^	1.02–1.08
Other	166,803	1,017,640	1839	1856.52	0.99	0.95–1.04
**GRADE**						
1	128,822	766,179	1488	1471.71	1.01	0.96–1.06
2	247,196	1,445,271	2494	2521.70	0.99	0.95–1.03
3 and undifferentiated	203,795	1,158,801	1822	1627.30	1.12^*^	1.07–1.17
Unknown	40,616	261,199	465	469.59	0.99	0.90–1.08
**ER**						
Positive^d^	461,616	2643512	4508	4574.82	0.99	0.96–1.01
Negative	112,254	633459	1110	878.50	1.26^*^	1.19–1.34
Unknown	46,559	354480	651	636.98	1.02	0.95–1.10
**PR**						
Positive^e^	395,138	2,250,962	3738	3819.13	0.98	0.95–1.01
Negative	172,749	978,035	1793	1544.46	1.16^*^	1.11–1.22
Unknown	52,541	402,454	738	726.70	1.02	0.94–1.09
**HER-2**^f^						
Positive	33,612	71,809	104	87.97	1.18	0.97–1.43
Negative	168,003	362,647	630	558.48	1.13^*^	1.04–1.22
Unknown	9,692	22,489	39	34.73	1.12	0.80–1.54
**BREAST CANCER SUBTYPE**^f^						
HER-2+/HR+	20,502	43,651	62	51.46	1.20	0.92–1.54
HER-2+/HR-	8,619	18,342	23	21.59	1.07	0.68–1.60
HER-2-/HR+	145,221	314,326	531	496.45	1.07	0.98–1.16
Triple negative	22,529	47,644	97	61.05	1.59^*^	1.29–1.94
Unknown	14,436	32,945	60	50.64	1.18	0.90–1.53
**MARITAL STATUS**						
Married	348,700	2,147,206	3066	3303.37	0.93	0.90–0.96
Not married^g^	244,878	1,346,809	2955	2549.36	1.16^*^	1.12–1.20
Unknown	26,851	137,436	248	237.56	1.04	0.92–1.18
**RADIOTHERAPY**						
Yes	315,604	192,442,616	3158	3186.04	0.99	0.96–1.03
None / Unknown	304,825	170,702,621	3111	2904.26	1.07	1.03–1.11
**SURGERY**						
Yes^h^	600,065	3,562,628	6123	5973.20	1.03	1.00–1.05
BCS	350,206	2,133,170	3712	3732.30	0.99	0.96–1.03
Modified radical mastectomy	141,202	912,291	1612	1489.69	1.08^*^	1.03–1.14
Total (simple) mastectomy	101,743	486,210	760	708.49	1.07	1.00–1.15
None	17,594	60,050	126	105.72	1.19	0.99–1.42
Unknown	2,770	8,774	20	11.37	1.76	1.07–2.72

a *Number of breast cancer survivors at the beginning of the study period*.

b *Begins 2 months after breast cancer diagnosed*.

c *Include American Incian/AK Native, Asian/Pacific Islander and others-unspecified*.

d *One patent with “only one side, side unspecified”; two patients with “paired site” were not include*.

e Include “borderline” and “positive.”

f *Did not include BC cases diagnosed before 2010 in SEER*.

g *Include divorced, separated, single, and widowed*.

h *39 cases received other types of mastectomy were not included*.

* *P < 0.05*.

SIRs were used to evaluate the incidence of second primary lung cancer in the cohort of patients with BC in relation to the expected incidence of lung cancer in the general population (Table 1). For the entire period, higher rates of second primary lung cancers occurred among BC survivors than in the general female population in SEER (SIR = 1.03; 95%CI: 1.00–1.06). SIRs were also high in black people and other people, and those who are unmarried. Lung cancer incidence rates were higher in more recent calendar years (SIRs for BC diagnosed in 2005–2009 and 2010–2014 were 1.05 and 1.13, respectively, and both *p* < 0.05). The SIR for lung cancer was significantly elevated within 1 year after BC diagnosed (SIR = 1.31; 95%CI: 1.23–1.39). SIR values decreased as the age at BC diagnosed rising, with the uppermost SIR reported for the youngest (age 20–39 years) cohort (SIR = 2.40; 95%CI: 1.75–3.23). Significant SIRs were also observed among infiltrating ductal carcinoma, the most common histological type, and poorly or undifferentiated histological grade. Hormone receptor and HER2 status are the most concerned aspects for breast cancer. Notably, we detected significant elevated SIRs among ER-, PR- and HER2- groups (SIRs were 1.26, 1.16, 1.13, respectively, all *p* < 0.05). Moreover, in the breast cancer subtype used in SEER, only TNBC group has a high incidence rate of lung cancer (SIR = 1.59, 95%CI: 1.29–1.94). No significant SIR was observed in BC patients who received radiotherapy. For BC patients after surgery, the SIR was high in “modified radical mastectiomy” group (SIR = 1.08; 95%CI: 1.03–1.14), but not in the breast conserving surgery (BCS) group or total (simple) mastectomy group.

Table 2 outlines demographic and clinicopathologic characteristics at BC diagnosis, grouped by subsequent lung cancer stage (353 cases were unstaged and thus not included here). Among the lung cancer stage groups, the distributions of stage at breast cancer diagnosis, laterality, histology were similar. Women with shorter latency and BC diagnosed at a more recent year more likely had an earlier stage of second lung cancer. This observation may reflect the much closer follow-up after initial BC diagnosed and the more common use of radiologic imaging in later years. On the country, patients among the ER-/HER2-/TNBC groups were more likely to have distant lung cancers. TNBC has a more aggressive clinical course than other types of breast cancer. Compared with other breast cancer, TNBC often develops visceral metastases. Those with TNBC had an increased likelihood of distant recurrence (13, 14).

**Table 2:** Patient and tumor characteristics at the time of lung cancer diagnosis.

**Breast cancers**	**Total (%)**	**Lung cancers**			***P value***
		**Localized (%)**	**Regional (%)**	**Distant (%)**	
Total	5916 (100)	1858 (31.4)	1590 (26.9)	2468 (41.7)	
**Latency**					< 0.001
≤ 1 year	915 (100)	421 (46.0)	267 (29.2)	227 (24.8)	
>1 year, ≤ 5 years	2495 (100)	770 (30.9)	674 (27.0)	1051 (42.1)	
>5 year, ≤ 10 years	1928 (100)	529 (27.4)	500 (25.9)	899 (46.6)	
>10 years	572 (100)	135 (23.6)	148 (25.9)	289 (50.5)	
Unknown	6 (100)	3 (50.0)	1 (16.7)	2 (33.3)	
**Race**					0.012
White	5109 (100)	1620 (31.7)	1359 (26.6)	2129 (41.7)	
Black	535 (100)	139 (26.0)	153 (28.6)	243 (45.5)	
Other	273 (100)	99 (36.3)	78 (28.6)	96 (35.2)	
**Age at breast cancer diagnosis**					< 0.001
20–39	40 (100)	12 (30.0)	10 (25.0)	18 (45.0)	
40–49	369 (100)	110 (29.8)	98 (26.6)	161 (43.6)	
50–59	1105 (100)	296 (26.8)	299 (27.1)	510 (46.2)	
60–69	2060 (100)	698 (33.9)	597 (29.0)	765 (37.1)	
70–79	1823 (100)	570 (31.3)	470 (25.8)	783 (43.0)	
80+	519 (100)	172 (33.1)	116 (22.4)	231 (44.5)	
**Year of breast cancer diagnosis**					< 0.001
2000–2004	3159 (100)	919 (29.1)	842 (26.7)	1398 (44.3)	
2005–2009	2018 (100)	651 (32.3)	545 (27.0)	822 (40.7)	
2010–2014	739 (100)	288 (39.0)	203 (27.5)	248 (33.6)	
**Stage at breast cancer diagnosis**					0.712
Localized	4288 (100)	1341 (31.3)	1165 (27.2)	1782 (41.6)	
Regional	1628 (100)	517 (31.8)	425 (26.1)	686 (42.1)	
**Laterality**					0.185
Right	3013 (100)	980 (32.5)	779 (25.9)	1254 (41.6)	
Left	2900 (100)	878 (30.3)	810 (27.9)	1212 (41.8)	
Unknown	3 (100)	0 (0.0)	1 (33.3)	2 (66.7)	
**Histology**					0.867
Infltrating ductal carcinoma	4170 (100)	1307 (31.3)	1129 (27.1)	1734 (41.6)	
Other	1746 (100)	551 (31.6)	461 (26.4)	734 (42.0)	
**Grade**					0.018
1	1416 (100)	481 (34.0)	352 (24.9)	583 (41.2)	
2	2348 (100)	748 (31.9)	651 (27.7)	949 (40.4)	
3 and undifferentiated	1715 (100)	488 (28.5)	466 (27.2)	761 (44.4)	
Unknown	436 (100)	141 (32.3)	121 (27.8)	174 (39.9)	
**ER**					0.030
Positive	4273 (100)	1381 (32.3)	1163 (27.2)	1729(40.5)	
Negative	1033 (100)	297 (28.8)	268 (25.9)	468 (45.3)	
Unknown	610 (100)	180 (29.5)	159 (26.1)	271 (44.4)	
**PR**					0.162
Positive	3543 (100)	1141 (32.3)	971 (27.4)	1431 (40.4)	
Negative	1681 (100)	511 (30.4)	547 (26.0)	733 (43.6)	
Unknown	692 (100)	206 (29.8)	182 (26.3)	304 (43.9)	
**HER-2**					0.031
Positive	101 (100)	39 (38.6)	34 (33.7)	28 (27.7)	
Negative	601 (100)	237 (39.4)	152 (25.3)	212 (35.3)	
Unknown	37 (100)	12 (32.4)	17 (45.9)	8 (21.6)	
**Breast cancer type**					0.021
HER-2+/HR+	73 (100)	31 (42.5)	23 (31.5)	19 (26.0)	
HER-2+/HR–	27 (100)	8 (29.6)	10 (37.0)	9 (33.3)	
HER-2–/HR+	509 (100)	207 (40.7)	132 (25.9)	170 (33.4)	
Triple negative	90 (100)	28 (31.1)	20 (22.2)	42 (46.7)	
Unknown	40 (100)	14 (35.0)	18 (45.0)	8 (20.0)	

**Figure 1:**
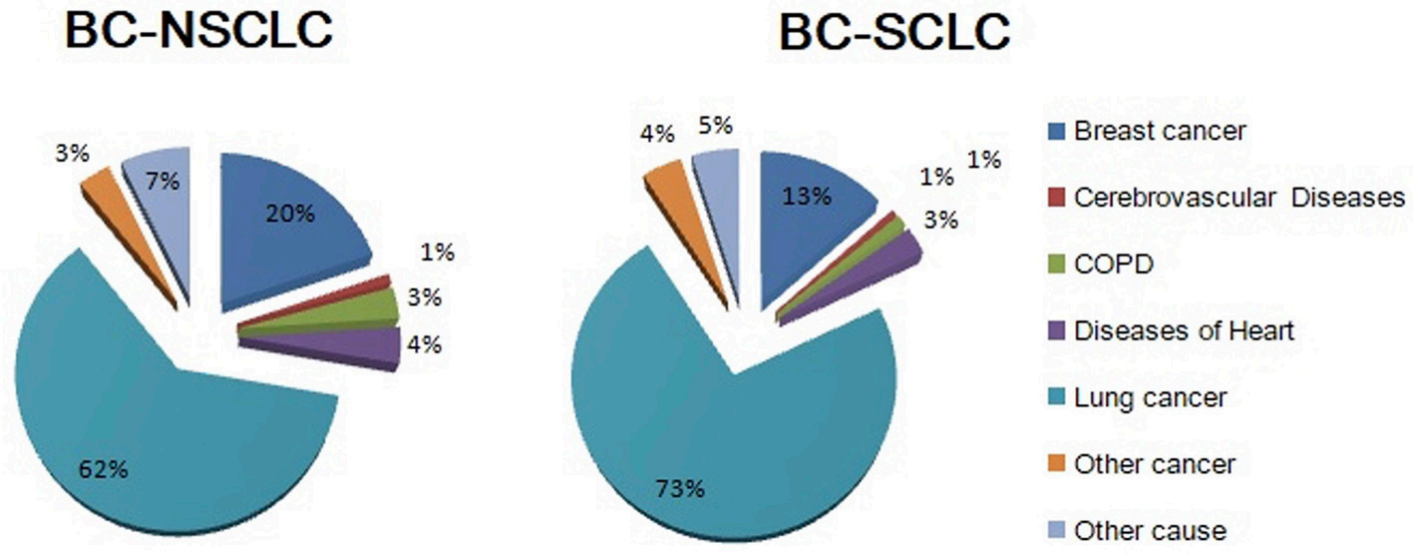
Cause of deaths grouped by histology among lung cancer patients who had a history of breast cancer.

Among the 6,269 breast cancer patients who had a subsequent primary lung cancer, 5,472 (87.3%) were NSCLC and 797 (12.7%) were SCLC. After a median follow-up of 52 months, 4,183 (66.7%) died. Figure 1 shows cause of death grouped by lung cancer histology. Mortality due to lung cancer accounted for most deaths (*n* = 2,654, 63.4%), and was observed to be greater among BC-SCLC (72.3%). Breast cancer is the second leading cause of death for both NSCLC and SCLC. Heart disease, chronic respiratory distress syndrome and cerebrovascular disease together accounted for a small number of deaths, comprising 8 and 5% of deaths for NSCLC and SCLC, respectively.

Table 3 shows the unadjusted OS of BC-NSCLC and BC-SCLC, along with survival of first primary NSCLC and SCLC, grouped by stage. OS was longer for BC-NSCLC vs. NSCLC-1 in each stage. Median survival (MST) after localized, regional and distant BC-NSCLC was 68.0, 26.0, and 6.0 m (47.0, 17.0, 5.0 m for NSCLC-1, respectively). Moreover, the survival rates were significantly higher for BC-NSCLC compared to NSCLC-1 in each stage. Five-year survival rates after localized, regional and distant BC-NSCLC were 53.9, 29.8, 5.7% and 44.4, 20.4, 3.0% for NSCLC-1 (all *p* < 0.001). MST for BC-SCLC (17.0, 15.0, and 5.0 m, in each stage respectively) was shorter versus BC-NSCLC. Compared to SCLC-1, the survival rates were no significant difference.

**Table 3:** Kaplan-Meier all-cause survival probabilities: women with lung cancer after breast cancer (BC-NSCLC and BC-SCLC) and first primary lung cancer (NSCLC-1 and SCLC-1).

**Stage**	**BC-NSCLC**	**SE**	**NSCLC-1**	**SE**	***P* value**	**BC-SCLC**	**SE**	**SCLC-1**	**SE**	***P* value**
**Localized**	***n*** **= 1,802**		***n*** **= 72,095**			***n*** **= 56**		***n*** **= 3,568**		
6-month survival	91.5%	0.7%	87.4%	0.1%	< 0.001	83.9%	4.9%	77.9%	0.7%	0.191
1-year survival	85.3%	0.9%	78.4%	0.2%	< 0.001	64.7%	6.6%	59.5%	0.8%	0.294
2-year survival	75.1%	1.1%	65.0%	0.2%	< 0.001	30.9%	6.7%	34.8%	0.8%	0.338
5-year survival	53.9%	1.4%	44.4%	0.2%	< 0.001	24.6%	6.7%	17.6%	0.7%	0.233
MST	68.0		47.0			17.0		16.0		
**Regional**	***n*** **= 1,376**		***n =*** **100,960**			***n =*** **214**		***n =*** **15,726**		
6-month survival	81.6%	1.1%	76.3%	0.1%	< 0.001	76.9%	3.0%	73.8%	0.4%	0.236
1-year survival	69.7%	1.3%	59.0%	0.2%	< 0.001	54.3%	3.6%	51.4%	0.4%	0.290
2-year survival	51.3%	1.5%	39.3%	0.2%	< 0.001	31.4%	3.5%	25.6%	0.4%	0.103
5-year survival	29.8%	1.5%	20.4%	0.1%	< 0.001	10.2%	2.8%	10.5%	0.3%	0.397
MST	26.0		17.0			15.0		13.0		
**Distant**	***n =*** **1,971**		***n =*** **221,634**			***n =*** **497**		***n =*** **51,646**		
6-month survival	49.1%	1.1%	43.9%	0.1%	< 0.001	44.5%	2.3%	48.5%	0.2%	0.089
1-year survival	31.6%	1.1%	24.9%	0.1%	< 0.001	20.1%	1.9%	22.9%	0.2%	0.136
2-year survival	16.6%	0.9%	11.1%	0.1%	< 0.001	7.5%	1.3%	6.5%	0.1%	0.297
5-year survival	5.7%	0.7%	3.0%	0.0%	0.0002	2.5%	1.0%	2.0%	0.1%	0.353
MST	6.0		5.0			5.0		6.0		

**Figure 2:**
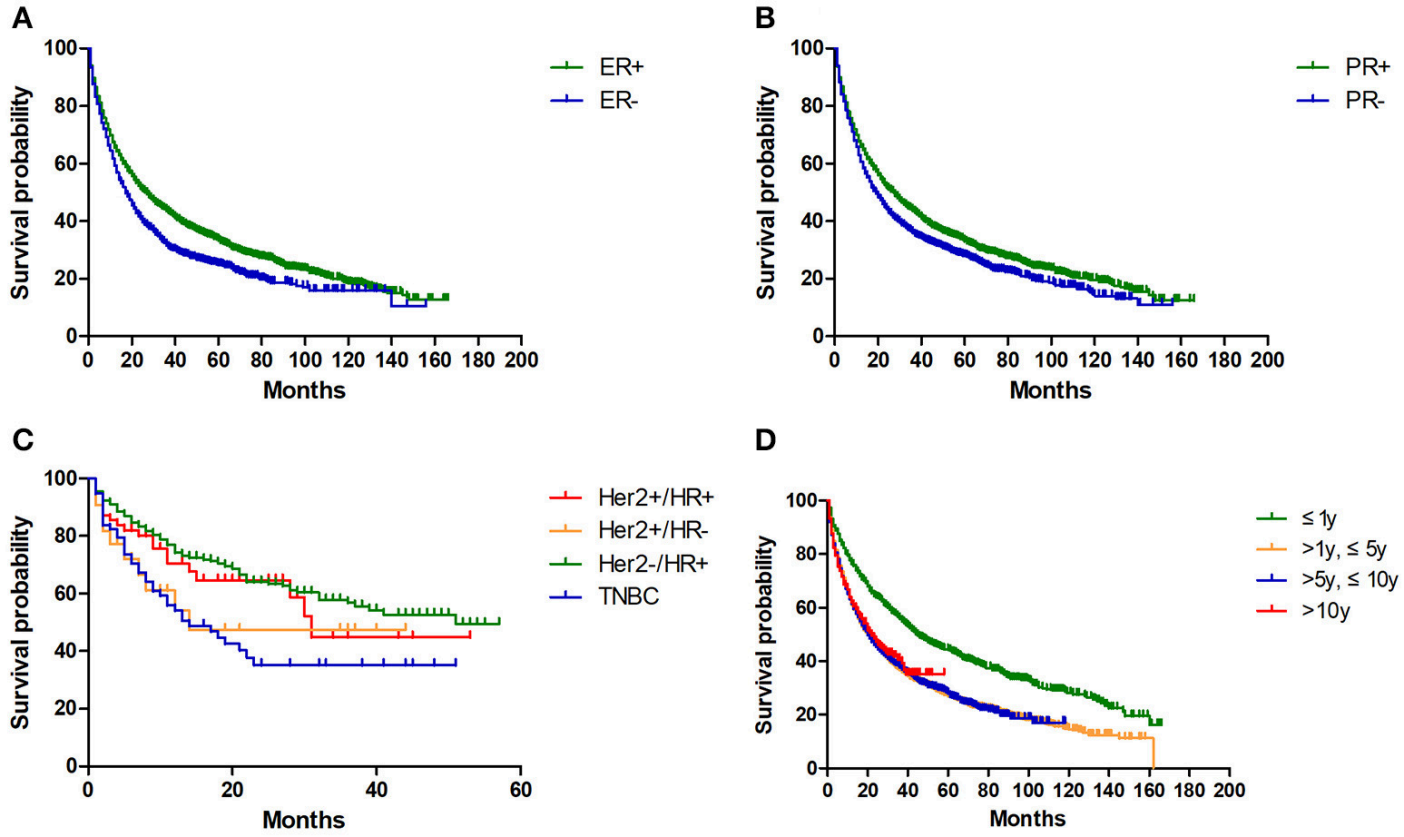
Kaplan-Meier overall survivals for BC-NSCLC patients, grouped by ER **(A)**, PR **(B)**, breast cancer types **(C)** and latency **(D)** (all *p* < 0.05).

Then we conducted log-rank test to evaluate potential variables affecting OS (Table 4). For BC-NSCLC, hormone status of breast cancer variables significantly impacted OS. As shown in Figure 2, BC-NSCLC patients with ER- (15.0 m vs. 22.0 m) and PR- (17.0 m vs. 23.0 m) had a shorter OS. MST for TNBC patients was only 13.0 months, which is the shortest compared to other breast cancer types (*p* = 0.001). Prolonged OS was observed in groups within 1 year after BC diagnosed. Favorable prognostic lung cancer variables for OS also included younger age at diagnosis, adenocarcinoma, earlier stage and lower grade. As for BC-SCLC, breast cancer characteristics did not significantly influence OS. BC-SCLC diagnosed at an earlier age or stage had a better prognosis.

**Table 4:** Survival analysis of second primary lung cancer after breast cancer.

**Variables**	**BC-NSCLC**, ***n =*** **5,416**				**BC-SCLC**, ***n =*** **794**			
	**Patients**	**Deaths**	**MST (mo)**	**Log-rank *P***	**Patients**	**Deaths**	**MST (mo)**	**Log-rank *P***
**BREAST CANCER VARIABLES**								
**Breast cancer stage**				0.380				0.620
Localized	3943	2509	20.0		548	462	8.0	
Regional	1473	950	22.0		246	203	9.0	
**ER**				< 0.001				0.316
Positive	3883	2371	22.0		582	482	8.0	
Negative	972	681	15.0		127	106	8.0	
**PR**				< 0.001				0.464
Positive	3210	1951	23.0		494	406	9.0	
Negative	1566	1059	17.0		208	175	7.0	
**HER-2**				0.626				0.589
Positive	92	34	31.0		10	4	15.0	
Negative	561	198	39.0		64	38	10.0	
**Breast cancer type**				0.001				0.550
HER-2+/HR+	67	24	30.0		7	3	15.0	
HER-2+/HR–	24	10	14.0		3	1	NA	
HER-2–/HR+	474	152	51.0		54	32	10.0	
Triple negative	85	46	13.0		10	6	12.0	
**LUNG CANCER VARIABLES**								
**Age at lung cancer diagnosis**				< 0.001				< 0.001
20–49	168	100	24.0		11	9	15.0	
50–69	2114	1203	29.0		346	275	10.0	
70+	3134	2156	15.0		437	381	6.0	
**Latency**				< 0.001				0.209
≤ 1 year	877	511	42.0		87	77	10.0	
>1 year, ≤ 5 years	2314	1629	17.0		318	274	9.0	
>5 years, ≤ 10 years	1712	1084	17.0		307	259	6.0	
>10 years	513	235	20.0		82	55	7.0	
**Histology**				< 0.001				NA
Adenocarcinoma	2910	1671	31.0		NA	NA	NA	
Squamous cell carcinoma	986	663	16.0		NA	NA	NA	
Others	1520	1125	11.0		NA	NA	NA	
**Lung cancer stage**				< 0.001				< 0.001
Localized	1799	744	68.0		56	39	17.0	
Regional	1376	824	26.0		213	162	15.0	
Distant	1971	1674	6.0		495	436	5.0	
**Grade**				< 0.001				0.253
1	520	168	92.0		1	1	6.0	
2	1135	543	49.0		2	1	7.0	
3 and undifferentiated	1407	989	17.0		234	205	9.0	

## Discussion

Over the past decades, survival after breast cancer diagnosis has improved throughout the world. With prolonged survival, there comes an increased likelihood that patients will receive a diagnosis of subsequent primary cancers as a result of underlying genetic or other risk factors related to breast cancer and treatment. In our large population-based study, we found that second primary lung cancer incident rates were modestly higher compared to general population. High-risk factors include: within 1 year after BC diagnosed, a young age at BC diagnosed, black people, poorly or undifferentiated histological grade of breast cancer, ER-/PR-/HER2- or TNBC. Second primary lung cancer is the predominant cause of death. NSCLC is the main histological type of secondary lung cancer. Survival rates for BC-NSCLC are much higher than first primary NSCLC in each stage. Compared to ER+/PR+ BC-NSCLC, ER-/PR- or TNBC correlate with a poorer prognosis. However, for BC-SCLC, the survival rates were no different to first primary SCLC. Breast cancer variables do not affect the survival of BC-SCLC patients.

Hormone receptor and HER2 status are very important for breast cancer. Notably, we found that they are correlated with incidence for second primary lung and survival of BC-NSCLC patients, especially for TNBCs. To date, no one has considered the risk of second primary lung cancer among TNBCs. Our study is the first to report that TNBCs have a much higher risk of secondary lung cancers. Epidermal growth factor receptor (EGFR) expression is reported in greater than 50% of TNBCs and correlates negatively with survival in these patients (15–17). Overexpression of EGFR in lung adenocarcinomas and TNBCs may provide one clue for common etiologic pathways (18, 19). Trails of EGFR inhibitors in breast cancer, however, have been disappointing. It remains to make a greater understanding of the molecular pathways (20).

The ER signaling pathway and lung cancer has been studied. Using the SEER database (1992-2008), Schonfeld et al. found that second lung cancer risks were significantly elevated among ER negative, but not ER positive breast cancer, which was consist with our results (21). As most of ER positive breast patients will receive antiestrogen therapy, a randomized trail studied the long-term effects on the incidence of secondary cancers of adjuvant tamoxifen. Patients received tamoxifen of 5 years had a significant lower incidence of second primary lung cancer compared to two-years of tamoxifen group (22). A study from Taiwan has followed up more than 6,000 breast cancer patients and 26 women developed second primary lung cancer. No difference of the relative risk of second lung cancer between those who have received anti-estrogens for breast cancer and those who have not was observed. However, second primary lung cancer patients who have received anti-estrogens had a significant longer survival time (10). An observational study from Switzerland got similar conclusion that compared with expected outcomes in the general population, breast cancer patients receiving anti-estrogen treatment had lower lung cancer mortality (23). Another retrospective population-based study from Canada suggests that antiestrogen use before and after the diagnosis of NSCLC is associated with decreased mortality (24). However, another large-scale clinical trial came to a different conclusion. In the post-intervention period of the Women's Health Initiative (WHI) trail, although estrogen plus progestin did not increase incidence of lung cancer in postmenopausal women, it increased the mortality of lung cancer, especially NSCLC (25). Nevertheless, the evidence above indicates that ER pathway plays an important role in lung cancer. The mechanism of ER signaling is through activation of EGFR/HER-1 and the IGF-1R pathways. An interaction between the ER and EGFR has been demonstrated in lung cancer cells (26–28). EGFR expression was down-regulated in response to estrogen and up-regulated in response to fulvestrant. Conversely, ERβ expression was down-regulated following treatment with EGF and up-regulated after treatment with gefitinib (27). The combination of anti-estrogen fulvestrant and an EGFR-TKI such as gefitinib or erlotinib can maximally inhibit lung cancer cell proliferation, induce apoptosis and reduce downstream signaling pathways both *in vivo* and *in vitro* (27, 29). A strong correlation between EGFR mutation and ER expression has been reported (30, 31). Therefore, these evidence provide a rational to use combined therapy (32, 33).

PR is an estrogen response gene. PR positive breast cancers are usually better differentiated tumors that respond to anti-estrogen therapy. PR positive was correlated with longer OS in NSCLC patients (34). Progesterone treatment inhibited the growth of lung tumor xenografts, and has also been shown to inhibitor invasion and migration of lung cancer cell lines.

In our study, lung cancer risks for ER+/PR+ BC patients are not significant compared to the general population. However, ER-/PR- BC patients have a higher incident rate of second lung cancer. Overall survival for ER+/PR+ BC-NSCLC patients were much longer compared to ER-/PR- BC-NSCLC patients. Moreover, we also found that the survival rates of BC-NSCLC were significant higher than first primary NSCLC patients. Although detailed information about individual endocrine therapy was absent, as most hormone receptor positive BC patients will receive endocrine therapy and the evidence listed above, our study implies some inhibitory effect of endocrine therapy on lung cancer carcinogenesis and progression.

HER2 deregulation has been described in lung cancer. The prognostic role of HER2 overexpression and HER2 amplification for lung cancer patients are controversial (35). HER2 mutations have been identified in ~1–6% NSCLC patients, Because of the low incidence, there's no enough data for defining whether HER2 mutation are prognostic in NSCLC (35). Tumor cells harboring HER2 mutations have been associated with response to EGFR-TKIs that target both EGFR and HER2 (e.g., afatinib, lapatinib) but not to those that target EGFR alone (36, 37). In HER2 amplified lung cancer xenograft models, combination of pertuzumab and ado-trastuzumab showed superior growth inhibitor to pertuzumab only (38). For HER2 positive NSCLC patients, enrollment of patients in clinical trials with novel agents targeting HER2 or downstream components of the MEK/ERK and PI3K/AKT/mTOR pathway is another option. As a relative small sample size of patients with known HER2 status in our study, the association between HER2 deregulation and second primary lung cancer after BC needs further study.

Radiotherapy plays an essential role in the treatment of early breast cancer, but is also reported to be associated with an increased risk of second malignancies after exposure. Postmastectomy radiotherapy for BC patients could sharply increase the risk of second primary lung cancer, especially in ipsilateral lung, among ever-smokers (8). After an analysis of the Longitudinal Health Insurance Database, Huang et al. found that the incidence of second primary lung cancer was higher in the radiotherapy group than in the non-radiotherapy group for BC patients (39). Another retrospective study from 12 U.S. population-based cancer registries also indicated an increased risk of second non-breast cancers (40). Grantzau et al. conducted a meta-analysis including 22 studies before August 1st 2013, which comprising 245,575 irradiated and 277,164 non-irradiated BC patients. For irradiated patients, the incidence of second cancers including lung cancer increased over time, peaking at 10–15 years after BC diagnosed (41). Subsequently, another study with meta-analysis of publications between 2010 and 2015 and population-based data also support the conclusion. Especially, for long-term smokers, the absolute risks of radiotherapy may outweigh the benefits (42). In our large population-based study using SEER data, no significantly high SIR was observed in the radiation group. However, nearly a half of BC patients are none/unknown status of radiotherapy, so this result may be biased and interpreted with caution. We also analyzed the risk of second primary lung cancer in BCS group, which is a surrogate marker for radiation. No significant SIR was detected. To further investigate the exact association between radiotherapy and second primary lung cancer, more detailed information of individual patients are needed to do stratified analysis or identify potential biomarkers.

There was a 31% increased risk of developing a second primary lung cancer within 1 year after breast cancer diagnosis, suggesting non-breast imaging studies are important during this period. Although all women with breast cancer experience an increased risk of second primary lung cancer, we found that younger women have a greater excess risk than the general population of the same age. Other studies also reported that the second cancer risk is substantially greater among patients with initial diagnosis of breast cancer at the age of < 50 years (6, 43, 44). Specially, we found that SIR values decreased as the age at BC diagnosed rising. BC patients diagnosed at 20–39 appear to have the highest SIR. So we compared the frequency distributions of ages at BC diagnosed according to ER/PR status using chi-square test (Supplementary Table S1). BC patients diagnosed at 20–39 are more likely to be ER-/PR- (both *p* < 0.001). Then we did stratified analysis according to ER/PR status. SIR values decreased as the age at BC diagnosed rising only in ER-/PR- groups, but not in ER+/PR+ groups (Supplementary Table S2). As mentioned above, this may be related to menstrual status and endocrine therapy. SIR of lung cancer increases in more recent years, likely reflecting the influence of advances in treatment and closer medical follow-up of BC survivors and also the improvement of screening methods. Black women with breast cancer tended to have greater risk of lung cancer. Racial differences in the risk of second malignancy after prostate cancer have been reported previously. It may reflect difference in genetic susceptibility or environmental exposures, treatment, or the combination of these factors.

From the aspect of breast cancer, second primary malignancies have a negative impact on survival (6). From the aspect of lung cancer, for the first time, our study indicated that a breast cancer history maybe a favorable prognostic factor for NSCLC (but not SCLC) patients. A previous study found that breast cancer history does not appear to adversely affect OS of NSCLC patients (11). Besides, our study is the first to evaluate the survival of second primary SCLC after breast cancer. BC histology does not influence the survival of SCLC patients. So, as mentioned above, the different effect of breast cancer histology to BC-NSCLC and BC-SCLC may contribute at least partly to the hormone receptor and HER2. Other common pathways between NSCLC and breast cancer deserve further study.

Large sample size is the great strength of our study, including 6,269 second primary lung cancers among 620,429 first breast cancers. Also, the SEER data were well collected and had extensive quality standards. The limitations include: (1) the lack of detailed individual information on treatment, such as endocrine therapy, targeted therapy. (2) absence of details of factors that could influence NSCLC survival, such as smoking, performance status, and sociodemographic status. (3) patients with lung metastases from breast cancer might have been misclassified as second primary lung cancers. In view of the improvements in histologically diagnostic procedures in the recent decades, and the rigorous criteria that SEER program requires to define multiple primary cancer (45), we estimate that any effects of residual misclassification are small.

In conclusion, the major finding of our study is that women diagnosed with breast cancer had an increased risk of developing a second primary lung cancer. Our study is the first to find that TNBCs, as well as ER-/PR- BC patients, have a significant high risk of secondary lung cancer. High-risk factors also include: short latency (within 1 year after BC diagnosed), younger age at BC diagnosed, black people, poorly or undifferentiated histological grade of BC. Although BC histology may be a favorable prognostic factor for BC-NSCLC patients, ER-, PR-, and TNBC are poor prognostic factors for them. ER, PR, and HER2 play an important role in the development and progression of BC-NSCLC. Therefore, we recommend closer medical follow-up of breast cancer patients with high-risk factors. For lung tumor after breast cancer diagnosis, biopsy may be very important to distinguish lung metastasis or second primary lung cancer, as the therapeutic regimens are largely different. Our findings should be replicated in other large, registry-based studies and prospective studies. Future researches are also need to address the clinical management and biologic mechanisms of lung cancer that develops after breast cancer.

## Author contributions

RW and ZY made substantial contributions to the design of the study, carried out the analysis, interpreted the data. LL contributed to the review of previous literature. WG and WL contributed substantially to the data discussion and critically commented on the manuscript for scientific content. YS and JX made substantial contributions to the conception and design of the study, data interpretation and drafting of the manuscript, were responsible for the quality of the overall manuscript. All authors approved the final version of the manuscript.

### Conflict of interest statement

The authors declare that the research was conducted in the absence of any commercial or financial relationships that could be construed as a potential conflict of interest.

## Abbreviations

BC: Breast cancer
NSCLC: non-small cell lung cancer
BC-NSCLC: second primary non-small cell lung cancer after breast cancer
BC-SCLC: second primary small cell lung cancer after breast cancer
NSCLC-1: first primary NSCLC
SCLC-1: first primary SCLC.
