# Supplementary material for: Second Primary Lung Cancer After Breast Cancer: A Population-Based Study of 6,269 Women
Source: Front Oncol. 2018 Oct 9;8:427. doi: 10.3389/fonc.2018.00427 (PMC6189405; doi:10.3389/fonc.2018.00427)
Supplement: Supplementary file 1 [file Table_1.DOCX]

**Supplementary Table S1** Frequency distributions of ages at BC diagnosed according to ER/PR status.

| **Age at breast cancer diagnosis** |  | ER | | | | PR | | |
| --- | --- | --- | --- | --- | --- | --- | --- | --- |
|  | total | | positive | negative | unknown | positive | negative | unknown |
| 20-39 | 44 (100%) | | 13 (29.5%) | 24 (54.5%) | 7 (15.9%) | 13 (29.5%) | 24 (54.5%) | 7 (15.9%) |
| 40-49 | 387 (100%) | | 249 (64.3%) | 105 (27.1%) | 33 (8.5%) | 225 (58.1%) | 127 (32.8%) | 35 (9.0%) |
| 50-59 | 1152 (100%) | | 747 (64.8%) | 275 (23.9%) | 130 (11.3%) | 623 (54.1%) | 384 (33.3%) | 145 (12.6%) |
| 60-69 | 2156 (100%) | | 1605 (74.4%) | 344 (16.0%) | 207 (9.6%) | 1320 (61.2%) | 585 (27.1%) | 251 (11.6%) |
| 70-79 | 1943 (100%) | | 1477 (76.0%) | 265 (13.6%) | 201 (10.3%) | 1218 (62.7%) | 504 (25.9%) | 221 (11.4%) |
| 80+ | 587 (100%) | | 417 (71.0%) | 97 (16.5%) | 73 (12.4%) | 339 (57.8%) | 169 (28.8%) | 79 (13.5%) |
|  |  | | *P*<0.001 | | | *P*<0.001 | | |
