# Supplementary material for: Second Primary Lung Cancer After Breast Cancer: A Population-Based Study of 6,269 Women
Source: Front Oncol. 2018 Oct 9;8:427. doi: 10.3389/fonc.2018.00427 (PMC6189405; doi:10.3389/fonc.2018.00427)
Supplement: Supplementary file 2 [file Table_2.DOCX]

**Supplementary Table S2** Stratified analysis of age at BC diagnosis according to ER/PR status.

| **Age at breast cancer diagnosis** | ER+ | | | | ER- | | | |
| --- | --- | --- | --- | --- | --- | --- | --- | --- |
|  | Observed | Expected | SIR | 95% CI | Observed | Expected | SIR | 95% CI |
| 20-39 | 13 | 19.72 | 1.21 | 0.65-2.07 | 24 | 5.43 | 4.42* | 2.83-6.58 |
| 40-49 | 249 | 197.21 | 1.26* | 1.11-1.43 | 105 | 57.74 | 1.82* | 1.49-2.20 |
| 50-59 | 747 | 754.29 | 0.99 | 0.92-1.06 | 275 | 201.49 | 1.36* | 1.21-1.54 |
| 60-69 | 1605 | 1568.57 | 1.02 | 0.97-1.07 | 344 | 301.29 | 1.14* | 1.02-1.27 |
| 70-79 | 1477 | 1503.53 | 0.98 | 0.93-1.03 | 265 | 236.46 | 1.12 | 0.99-1.26 |
| 80+ | 417 | 540.5 | 0.77* | 0.70-0.85 | 97 | 76.09 | 1.27* | 1.03-1.56 |
| **Age at breast cancer diagnosis** | PR+ | | | | PR- | | | |
|  | Observed | Expected | SIR | 95% CI | Observed | Expected | SIR | 95% CI |
| 20-39 | 13 | 9.46 | 1.37 | 0.73-2.35 | 24 | 6.49 | 3.70* | 2.37-5.50 |
| 40-49 | 225 | 178.65 | 1.26* | 1.10-1.44 | 127 | 72.94 | 1.74* | 1.45-2.07 |
| 50-59 | 623 | 630.21 | 0.99 | 0.91-1.07 | 384 | 309.57 | 1.24* | 1.12-1.37 |
| 60-69 | 1320 | 1302.01 | 1.01 | 0.96-1.07 | 585 | 532.56 | 1.10* | 1.01-1.19 |
| 70-79 | 1218 | 1252.7 | 0.97 | 0.92-1.03 | 504 | 460.13 | 1.10* | 1.00-1.20 |
| 80+ | 329 | 446.1 | 0.76* | 0.68-0.85 | 169 | 162.76 | 1.04 | 0.89-1.21 |

******P* < 0.05.
